# Considerations for Domestication of Novel Strains of Filamentous Fungi

**DOI:** 10.1021/acssynbio.4c00672

**Published:** 2025-01-30

**Authors:** Randi M. Pullen, Stephen R. Decker, Venkataramanan Subramanian, Meaghan J. Adler, Alexander V. Tobias, Matthew Perisin, Christian J. Sund, Matthew D. Servinsky, Mark T. Kozlowski

**Affiliations:** † DEVCOM Army Research Laboratory, 2800 Powder Mill Rd., Adelphi, Maryland 20783, United States; ‡ National Renewable Energy Laboratory, 15013 Denver West Parkway, Golden, Colorado 80401, United States

**Keywords:** synthetic biology, filamentous fungi, molecular biology, microbial cell factory

## Abstract

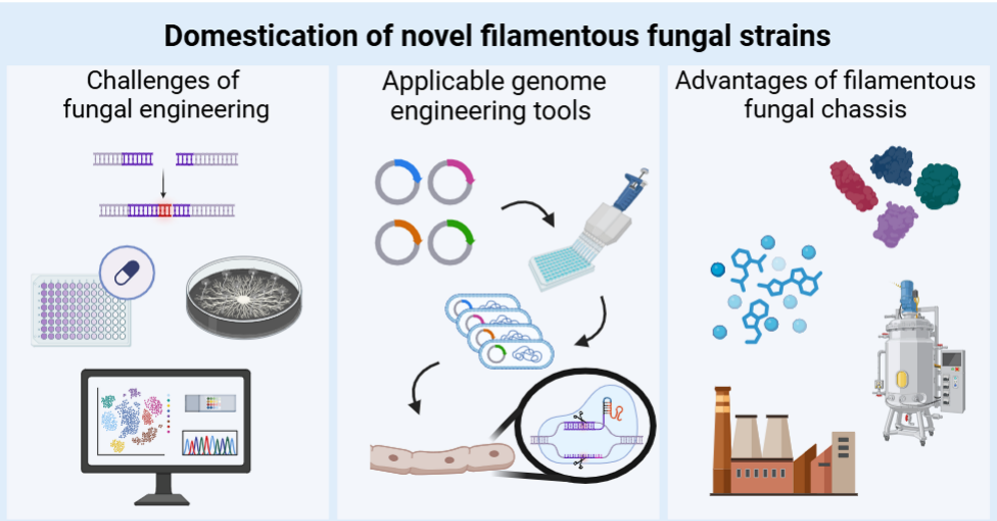

Fungi, especially filamentous fungi, are a relatively understudied, biotechnologically useful resource with incredible potential for commercial applications. These multicellular eukaryotic organisms have long been exploited for their natural production of useful commodity chemicals and proteins such as enzymes used in starch processing, detergents, food and feed production, pulping and paper making and biofuels production. The ability of filamentous fungi to use a wide range of feedstocks is another key advantage. As chassis organisms, filamentous fungi can express cellular machinery, and metabolic and signal transduction pathways from both prokaryotic and eukaryotic origins. Their genomes abound with novel genetic elements and metabolic processes that can be harnessed for biotechnology applications. Synthetic biology tools are becoming inexpensive, modular, and expansive while systems biology is beginning to provide the level of understanding required to design increasingly complex synthetic systems. This review covers the challenges of working in filamentous fungi and offers a perspective on the approaches needed to exploit fungi as microbial cell factories.

## 1 Introduction

Filamentous fungi are ubiquitous in nature and interact with a vast array of organisms and materials of natural and anthropogenic origin.^1−4^ Interspecies interactions run the gamut from pathogenic to mutualistic, with examples from agriculture where fungi can either decimate or enhance crop yields.^5−9^ Interactions with natural materials contribute to global-scale processes, such as unlocking carbon from decaying plant matter in the carbon cycle.^10^ Beyond the natural world, filamentous fungi contribute to deterioration of an array of materials such as concrete, glass, and metals.^4,11,12^ The ubiquity and diversity of filamentous fungi indicate they have evolved systems to thrive in a multitude of environments. Despite global importance, researchers are only beginning to understand filamentous fungi, with only a small percentage of the organisms identified. Within these organisms, many of the genes have unknown functions.^13−18^ Thus, the genomes of filamentous fungi are a largely untapped resource of information about how to interact with, produce, and transform materials across the world.

Humans have a long history of using native, unmodified fungi to produce food.^19^ Yeasts garner the most recognition due to their roles in bread and alcoholic beverage production, but unmodified filamentous fungi also have a major impact. In addition to mushrooms and truffles consumed directly, molds have been used in the production of fermented and ripened foods.^19^ The primary contribution of molds to food fermentation is the secretion of a wide range of catabolic enzymes including amylases, proteases, and lipases.^19^ These enzymes break down the food substrate, with the products released moderating the natural consortia made up of bacteria and yeasts which, in total, contribute to the overall desired properties of the food.^20,21^ One of the oldest processes is the generation of koji, a culture of *Aspergillus oryzae* and/or *Aspergillus sojae* grown on rice, grains, or tubers.^19^ The resultant mash contains a complex mix of lytic enzymes which act on various food products to produce a range of fermented goods such as shoyu (soy sauce), miso, fermented bean pastes, rice wine, and sake.^22^ Rind-ripened and blue-veined cheeses are produced using *Penicillium camemberti* and *Penicillium roqueforti*, respectively.^23^ A variety of *Penicillium* species are involved in curing sausages and meats as well.^24^ Fungal-hyphae-based meat substitutes have recently been developed, with several companies growing, processing, and aligning filamentous fungal hyphae to simulate a variety of meats.^25^

Over the last century, filamentous fungi have been used to produce a diverse range of products including enzymes, proteins, and metabolites.^26,27^*Aspergillus oryzae* was the first fungal strain specifically developed as a targeted enzyme producer. In 1894, Takadiastase was produced specifically for market using a koji-derived strain of *A. oryzae.*(27) Originally marketed as a digestive aid, the commercial development of this amylase kicked off an industrial enzyme revolution as it led to the discovery that amylases and related enzymes could completely break down starch into glucose, while avoiding transglycosylation and the generation of high levels of salt inherent in the then-standard acid hydrolysis process.^27^ By the 1960s, the entire starch-to-sugar industry had switched from acid to enzyme-based sugar production, and a great deal of research was being carried out on multiple *Aspergillus* species, including *Aspergillus niger*, to develop better enzymes for the starch hydrolysis market.^27^ During this same period, James Currie discovered that *A. niger* grown under the right conditions would produce and secrete large amounts of citric acid.^26^ His selection of a high-productivity strain combined with a systematic optimization of substrate, nitrogen concentration, and pH conditions was the first concerted effort to maximize a commercial fungal product.^27^ This discovery, made in 1917, was rapidly developed and patented by Pfizer, who hired Currie as their first research chemist and then built its first pilot plant for citric acid by fungal fermentation. By the late 1920s, Pfizer was producing 10 M lbs of citric acid by this process annually and dominated the entire market.^28^

Modern industrial fungal enzyme production includes a wide array of activities. Industrial fungal enzymes, such as cellulases from *Trichoderma reesei*, play major roles in deconstruction of structural polysaccharides in plant cell walls.^29−31^ As plants comprise around 90% of the biomass-based carbon on the planet, it is unsurprising that fungi have evolved a highly complex and adaptable suite of enzymes targeting this vast sugar reservoir.^10^ Many fungal-produced enzymes are industrially important, and fungal strains have been selected, crossed, and genetically engineered to produce these enzymes for use in starch processing, detergents, food and feed production, pulping and paper making, biofuels production, as well as pharmaceutical and chemical industries.^29−37^

Humans are only beginning to harness the amazing complexity and diversity of fungal biology. Filamentous fungi pose unique challenges compared to unicellular organisms such as yeast and bacteria. These challenges include the technical difficulties in transforming fungal cells, diverse morphologies and life states of filamentous fungi, lack of selectable markers, and lack of robust genome database resources.^13,14,38−47^ Funding of foundational research toward biomanufacturing is largely driven by health and medicine, with researchers frequently preferring to use common tractable yeast and bacterial strains. Given the history of filamentous fungi revolutionizing industry, the ravine that currently lies between foundational fungal research and modern biomanufacturing should be bridged through synthetic biology. While certain organisms such as *Aspergillus nidulans, Neurospora crassa*, *A. niger* and *A. oryzae* have been domesticated, there are many other potentially useful species such as *T. reesei* whose domestication is not yet complete. Chassis organisms capable of surviving harsh conditions such as low pH, high salt, high temperatures, or large amounts of radiation would be needed for operations in difficult conditions, such as remediation of mining wastes, or outer space. The rapid deployment of state-of-the-art tools in metabolic engineering, genome manipulation, protein engineering, expression control, and strain development can be leveraged to expand the arsenal of fungal chassis organisms, providing new platforms for biomanufacturing of important commodity products, recycling of wastes, and discovery of new biosynthetic routes. In this review, we will briefly discuss the challenges of domesticating novel fungi, and briefly show how those challenges were overcome in certain chassis strains.

## 2 Challenges of Filamentous Fungi as Chassis

The past decade has seen huge advances in modular vector designs and technologies for working in yeast with many reviews covering the topic,^48−51^ however, capabilities for the majority of filamentous fungi have lagged until very recently. This delay is largely due to the increased complexity of these tough multicellular organisms and the resulting complications in experimental design. Several *Aspergillus species* (notably *A. niger, A. awamori, and A. nidulans*) and *N. crassa* have emerged as chassis and model strains in filamentous fungal molecular genetics, with a full suite of genetic engineering tools available for these microbes.^52^ Other genera, such as *Trichoderma, Penicillium, Myceliopthera, Fusarium*, and others have been engineered for various protein and bioproduct expression, however, these genera can have problems with nonhomologous end joining (NHEJ), and syn-bio tools such as auxotrophic mutants, CRISPR/Cas9, Golden Gate cloning, high efficiency transformation, selection markers, specific promoters and cloning vectors, and signal sequences for these and other species are not well established, limiting development and utilization of these organisms.^37^ Even with recent demonstrations of vector design improvements, CRISPR/Cas9 gene-editing technologies, and transformation methods, more work is needed to fully understand and control genetic manipulations in filamentous fungi.^38^ While filamentous fungi have been used extensively for the production of foods, pharmaceuticals, enzymes, and other vital products, only a handful of species are used, and many species are not yet genetically tractable.^53^ There are an estimated 6 million fungal species,^54^ and domesticating more of them as chassis species would enable fungi to be used in a wider range of growth conditions, such as higher temperatures or salinity, or using very specific organic substrates as carbon sources most efficiently without the need for extensive metabolic engineering. Therefore, our review intends to highlight the current challenges that must be overcome to bring up a robust filamentous fungal chassis, and briefly discuss how these challenges were solved in *A. nidulans, N. crassa*, and *A. oryzae*, which are at-present widely used and modified filamentous fungi.

### 2.1 Nonhomologous End Joining

The approaches normally used for targeted genome integration rely on homologous recombination (HR) in the target organism. However, the dominant mode of DNA repair in filamentous fungi is nonhomologous end joining (NHEJ), with 80% of repairs of double-strand breaks in filamentous fungi occurring via the NHEJ mechanism.^55,56^ Low frequencies of HR in some fungal species mean that obtaining integration of transforming sequences by homologous recombination necessitates the use of extended sequences in the transforming DNA that are highly homologous or, preferably, identical to the target genome sequences.^57,58^ Combined with highly variable transformation efficiencies for different fungi,^40^ this makes it difficult to obtain large populations of transformants for some species. This in turn can make it difficult to screen large numbers of genome modifications. Therefore, improving the frequency of HR is the first major challenge to establishing a system for targeted genetics in new fungal species. Deletion or knockdown of fungal homologues of the human *Ku70*, *Ku80*, and DNA ligase IV genes, which are major components of the NHEJ pathway, generally leads to improved targeted genome integration.^59,60^ The majority of NHEJ deletion strains are found in *Aspergillus* and *Neurospora*, though targeted knockout or knockdown of these NHEJ pathway components has boosted HR efficiencies to 60–100% across several species.^41,59,61−70^

Deletions of Ku homologue genes can sometimes make organisms more sensitive to mutations caused by chemicals or UV,^71−75^ and mutation can have a cumulative effect on the viability of the fungus.^76^ Growth arrest and accelerated shortening of telomeres has also been observed in certain fungi following deletion of Ku homologues.^77,78^ However, in some organisms such as those of genus *Aspergillus*, the Ku deletions do not actually appear to harm organismal viability, due to the presence of redundant DNA repair machinery in these organisms.^79,80^ Therefore, while deletion of Ku homologues frequently solve the NHEJ problem, the side effects of doing so will likely vary from organism to organism, and the domesticator of an organism should be prepared to test for defects in growth and increases in mutations following deletion of those genes. As an alternative to deleting NHEJ pathway genes, transient knockdown, post-translational control, or controlled expression of those genes can also improve the frequency of HR. Whereas short lengths of homologous regions (30–50 bp) are sufficient to cause HR in yeasts such as *Saccharomyces cerevisiae*, several hundred base pairs are required for HR in higher fungi, a consideration for vector design.^41,63,81,82^

### 2.2 High-Throughput Screening

High-throughput screening of microorganisms depends on the availability of single colonies or cells that can be subjected to individual testing by picking colonies from agar plates into microtiter plates and/or using techniques to screen single cells in their suspended form. For certain species, it can be challenging to obtain single spore colonies using classical approaches such as colony screening on agar plates or growth in microtiter plates. The hydrophobicity of many spores, as well as the presence of spore production structures such as conidiophores and sporangia, result in aggregated spores that can be difficult to separate: this problem is usually solved by the addition of surfactant. In certain species, particularly fast-growing fungi, spreading mycelia can rapidly cover the entire surface of the agar in a Petri dish, so colony restricting agents such as detergents^83^ or Rose Bengal^84^ are often used to retain single colonies. Some specialized screenings have been developed for pathogenic fungi using classical microtiter plate-based methods as well as novel lab-on-a-chip technology.^85^

**Figure 1:**
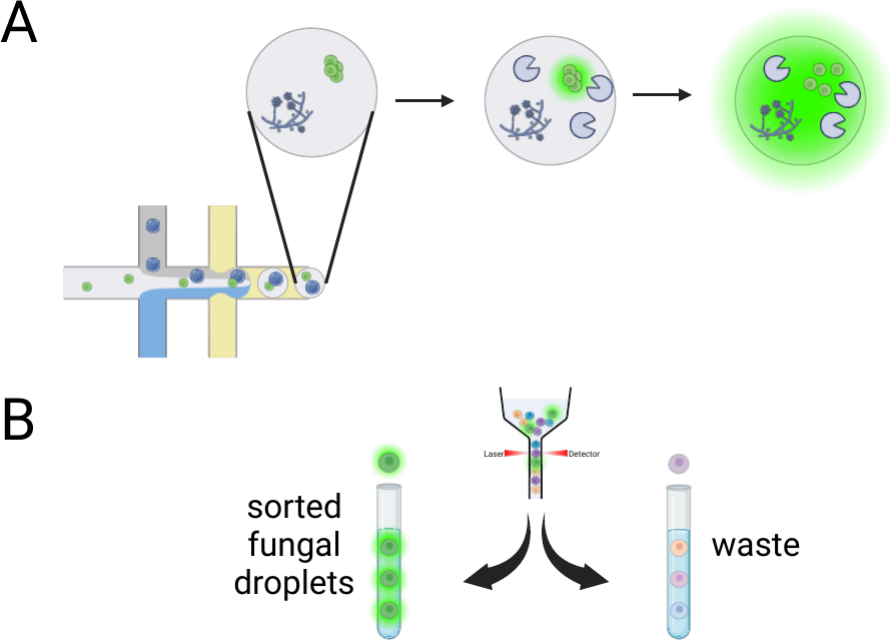
Fluorescence activated droplet sorting (FADS) of fungi. (A) Droplets of a fungal spore library also contain a fluorogenic enzyme substrate, fluorescence being quenched in the native state. As the spores germinate and grow, secreted enzymes digest the substrate. (B) Droplets are loaded for sorting with enzyme activity corresponding to fluorescence intensity. Figure created with Biorender.com.

Filamentous fungi are not easily amenable to fluorescence activated cell sorting (FACS), unlike most bacteria, yeasts, and some eukaryotic cells. The difficulties stem from the incompatibility of long hyphal chains with FACS fluidics. To circumvent this issue, Beneyton et al. germinated *A. niger* conidia in nanoliter-size droplets that they then subjected to fluorescence activated droplet sorting (FADS) based on enzyme production (Figure 1).^86^ This was a milestone achievement for enabling high-throughput screening and enrichment of production strains of filamentous fungi that far surpasses microtiter plate-based screening in terms of throughput. A major benefit of droplet-based sorting is the ability to screen for secreted products. He et al. demonstrated with *T. reesei* that the droplet preparation, germination rate, droplet size, detection settings and sorting are all parameters that must be finely tuned for a robust FADS platform.^29^ We expect that recent improvements in droplet-based sorting will lead to a large increase in the utilization of this approach for screening large and diverse libraries of filamentous fungi generated by random or combinatorial methods.^87−89^

### 2.3 Selectable Markers

Selection markers play an important role in synthetic biology due to their application in gene knockouts and knock-ins. This is particularly the case with organisms which require multiple gene deletions to enable development into a robust expression host, such as *T. reesei.* Auxotrophic markers such as *argB* (ornithine carbamoyltransferase), *ura3*/*pyr4*/*pyrG* (orotidine-5′-phosphate decarboxylase), *pyr4* (OMP decarboxylase), *ura5* (orotate phosphoribosyltransferase), *ade2* (phosphoribosylaminoimidazole carboxylase), and *hxk1* (hexokinase) have been developed for *T. reesei.*(41−44,90,91) This is particularly useful for recalcitrant strains such as *A. oryzae*, which has a high resistance to common antibiotics and only a small handful of available marker genes: *sC* (ATRP sulfurylase),^46^*niaD* (nitrate reductase),^92^*pyrG,*^93^ and *thiA* (pyrithiamine resistance).^94^ Unlike the commonly used markers, Todokoro et al. discovered a new pyrithiamine resistance gene, *thiI*, which does not cause auxotrophy or growth defects.^95^

Dominant markers provide an advantage over auxotrophic markers as they do not require development of an auxotrophic mutant. In this realm, antibiotic selection markers such as *hph* (hygromycin B phosphotransferase), *ble* (zeocin resistance), *npt2* (sodium/phosphate cotransporter), and *ptrA* (pyrithiamine resistance gene), the nitrogen utilizing marker *amdS*, and the sucrose utilizing marker *suc1* have been developed for *T. reesei*.^45,91,96−98^ Other selection markers such as *bsd* (blasticidin S deaminase), *bar* (glufosinate resistance), and *sC* (ATRP sulfurylase) have been tested in other fungal systems.^46,59,99^ Multiple gene modifications would require the use of multiple selectable markers or multiple rounds of recycling of the same marker. While selectable markers are integral to verifying genetic modifications, maintaining selective pressure is a major obstacle for scale-up production. The development of recyclable markers such as *pyr4*, along with the availability of the Cre/*loxP* recombination system provides added flexibility to improving sequential gene deletions and eliminating the maintenance of special feedstocks to maintain selective pressure.^47,90,100^

### 2.4 Databases and Modeling

Fungal synthetic biologists have access to a few public genomic databases dedicated to fungi at their disposal. FungiDB is a database currently containing genomes for 319 fungi.^13^ Features include a genome browser, a CRISPR guide RNA design tool, and many types of searches (transcriptomic, proteomic, metabolomic, etc.). The MycoCosm database contains over 2000 fungal genomes.^14^ Each genome in MycoCosm has its own organism portal, which provides access to sequence data as well as the tools available for use with that organism. The tools in MycoCosm are similar to those in FungiDB. Ensembl Fungi^15^ is the third major fungal genome database. It currently contains 1505 fungal genomes^14^ and its features are focused on comparative genomics.

The fungal genome databases above can be a rich resource for fungal scientists and engineers looking for homologues of known enzymes, sequence information for designing PCR primers, or homology arms for genome editing DNA cassettes for use with a particular species. On the other hand, the genomes in these databases tend to be sparsely annotated, even for well-known and popular fungi. A large percentage of algorithm-identified ORFs are labeled as “hypothetical” genes and more detailed annotations and functional predictions are not necessarily validated. The dearth of annotations limits the utility of these genomic resources, especially for constructing genome-scale metabolic models, whose accuracy depends on highly complete and accurate annotation of metabolic genes.^53^ Fungal genes and genomes are also underrepresented in public databases. This is a function of the paucity of laboratories devoted to fungal research and the resources available to them. For example, the number of genomes available in the NCBI RefSeq collection of annotated, nonredundant reference sequences is 398 for fungi compared with 37,232 for bacteria.^16^

Improving the state of public fungal genomic databases will require substantial resources and likely growth of the global fungal research community. In addition to this, we believe that improvement of fungal genomic resources can be accelerated by taking a more community-based approach that begins with utilization of simple assembly and annotation pipelines by individual laboratories. After submission of these first-draft annotated genomes to a public repository, the genome may be processed automatically by additional, more sophisticated annotation algorithms prior to being posted. Subsequently, it should be easy for members of the community to add their own annotations and notes to posted genomes with a Wiki-type system and interface. This type of interactive philosophy and implementation is exemplified by KBase,^102^ an open platform for systems biology modeling established by the U.S. Department of Energy. KBase allows users to upload data, analyze it alongside collaborator and public data, build and iterate their own models, then share and publish their workflows and conclusions. More-extensive databases will have positive implications for both synthetic and systems biology, as knowing what the genes are will enable researchers to better understand how they are linked, or how they might be expressed heterologously in another organism.

### 2.5 Production of Toxins

The production of toxins as secondary metabolites is also something experimenters should be aware of when attempting to domesticate new strains. The number of known toxic metabolites is increasing with characterization of novel fungal genomes, revealing more areas of potential concern.^103^ For example, the T-2 class of type A trichothecene mycotoxins produced by *Fusarium* and aflatoxins produced by *Aspergillus* are of great concern to human health.^104−107^ T-2 toxins are non-nitrogenous compounds that target eukaryotic protein synthesis, and cause chronic acute toxicity and induced apoptosis in immune system cells.^108^ T-2 toxin is produced by 10 biosynthetic genes referred to as the Tri5 gene cluster, which is conserved across many species.^109^ These fungal toxins are often very resilient, and harsh chemical and physical methods, such as heating to 250 °C or application of alkylating agents, can be required to deactivate them.^8,105^ Toxic secondary metabolites can also be removed using enzymes, such as removing ochratoxin A (OTA) by use of amido-hydrolases or ochratoxinase.^8^ Culture filtrates of probiotic fungi can also be used in conjunction with culture of fungi that may produce dangerous metabolites, in order to remove those dangerous metabolites.^105^ Nonmycotoxin-producing bacterial and fungal strains can also be introduced in coculture with fungi of concern, in order to limit mycotoxin production.^104,105^ Alternatively, large chromosomal deletions can be generated using the loop-out recombination technique that can enable removal of gene clusters involved in secondary metabolite synthesis.^110^ The most extensive removal of secondary metabolite gene clusters has been carried out by the Oakley and Wang laboratories, who were able to remove a quarter of a million base pairs.^111,112^ This demonstrates that large toxic secondary metabolite clusters can be removed, provided that the experimenter knows where the clusters are.

Identifying and cataloging novel secondary metabolites is therefore critical to determine whether a candidate chassis organism poses a health hazard, and if it does, which genes should be deleted before it is pressed into wider use. Once these hazardous metabolites have been identified, they can be removed by CRISPR or other gene editing methods, as reviewed by Jiang et al.^113^ Besides direct gene editing, epigenetic control and regulation can also be used to reduce the production of mycotoxins via secondary metabolite pathways.^114,115^

### 2.6 Currently Domesticated Filamentous Fungi: *A. nidulans*, *N. crassa*, and *A. oryzae*

The challenges of domestication and toolkit development outlined in the previous sections, particularly NHEJ, selectable markers, and the creation of genomic databases, have been solved to considerable extent in the workhorse organisms *A. nidulans, N. crassa*, and *A. oryzae*. In this section we discuss the steps taken to domesticate these organisms, which we hope will serve as a starting place for those who wish to domesticate other strains of filamentous fungi that may be attractive for use in extreme or unusual environments.

#### 2.6.1 *Neurospora crassa*

*Neurospora crassa* is a well-known and extensively utilized filamentous fungus, used to investigate cell polarity and fusion, pathogenic mechanisms such as secretion, and circadian rhythms,^116−121^ and its range of applications continues to expand, such as becoming a host–virus model.^122^ The major breakthroughs required for the domestication of *N. crassa* were: effective selection cassettes/markers, highly efficient knockout mutant and transformation protocols, and the generation of robust -omics resources. Auxotrophic biochemical mutants of *N. crassa* were developed as early as 1944, and auxotrophic strains have been used extensively since.^116,121^ Of particular interest is the “one-gene, one-protein” model that won the Nobel Prize in Physiology or Medicine for Beadle and Tatum in 1958.^123−125^ The early development work of *N. crassa* into a model organism is reviewed by Roche and co-workers.^121^ The history of the exploration of the *N. crassa* genome is extensively reviewed elsewhere.^126,127^

Dominant selective markers have a long history in *N. crassa,* seen in Austin et al. 1990, where the bacterial *ble* gene was transitioned, conferring resistance to bleomycin into *N. crassa*, specifically to support the use of strains without auxotrophic markers.^128^ Other dominant selection markers used in *N. crassa* include resistance genes against antibiotics such as phosphinothricin and hygromycin as well as the *amdS* gene from *Aspergillus*, which enables acetamide to be used as a sole nitrogen source.^116,128−132^ The time and energy spent to characterize minimum inhibitory concentrations (MIC) for various mutant strains and wild strains can be considerable when making a new chassis organism.

*N. crassa* was the first filamentous fungus to successfully have a genetic modification.^120^ After 30 years of investigation, the problem of NHEJ was solved by knocking out the DNA repair genes *mus51* and *mus52*, which are homologues of Ku70 and Ku80.^133^*Mus51*- and *mus52*-knocked down strains showed 100% of transformants with successful targeted integration compared to the wild-type strain with 10–30% of transformants. This advancement of efficient targeted integration of cassettes, in combination with the well-characterized mating biology of *N. crassa*, supported combinatorial knockout strain construction via mating crosses with other knockout strains or mating crosses with the parental wild-type strain under selection for the inserted cassette, thereby removing the NHEJ knockdown feature. This knockout strain has been used extensively in the literature.^120,131,132,134^ Ishibashi and co-workers investigated the impact of the *mus51* or *mus52* knockouts on the transcriptional landscape of *N. crassa*, showing modulation in different extracellular inorganic phosphate conditions, including upregulation of another NHEJ DNA repair gene *mus53*, which is homologous to the human gene LigIV.^135^ Fungal transformation using different approaches such as spore electroporation, biolistic transformation, PEG-mediated transformation, and *Agrobacterium*-mediated transformation (AMT) have been optimized for three model fungal species *A. niger, T. reesei,* and *N. crassa*(136−138)

Finally, work in *N. crassa* has been supported by a well-annotated genome created by extensive functional genomics studies. This information is neatly collated in the Neurospora Genome Project hosted at the Dunlap and Loros laboratories at the Dartmouth Geisel School of Medicine.^120,139^ The Neurospora Genome Project contains ORF knockout collections, SNP primer sets, and detailed public protocols for developing knockout strains in-house via genetic tools and traditional mating crosses (https://geiselmed.dartmouth.edu/dunlaploros/genome/). Many of the applications of the *N. crassa* model draw on the depth of genetic data available.^122,140,141^

However, because *N. crassa* was one of the first organisms to be domesticated, many historical strains are used. Over time, these historical strains have diverged from each other and formed secondary mutations of which the experimenters may not be aware. Secondary mutations have been shown to be responsible for morphology phenotypes that influence polarized growth.^119^ Newly domesticated strains may not have this issue, as there has been less time for their genotypes to diverge from lab to lab. In summary, *N. crassa* is regarded as a model organism for higher eukaryotic molecular biology with a comprehensive genomic database. *N. crassa* showcases the value of characterized selection markers, NHEJ knockdown approaches for rapid genetic engineering, and the caution needed for using historical strains.

#### 2.6.2 *Aspergillus oryzae*

*Aspergillus oryzae* is one of the most widely used industrial multicellular fungi, primarily involved in the production of fermented foods such as miso (soybean paste), shoyu (soy sauce), tane-koji (seed rice malt), douchi (fermented and salted black soybean), bean curd seasoning and vinegar. This versatility has been primarily attributed to its ability to produce amylase and protease enzymes that can convert starch and proteins into simpler sugars and amino acids. In addition, *A. oryzae* is also known for production of secondary metabolites such as kojic acid, L-malic acid, and salidroside, and also has a higher number of secondary metabolite gene clusters in comparison to other sequenced species.^142−148^ Heterologous secondary metabolite pathway genes have also been expressed in this organism.^149,150^ This fungus also possesses a robust protein expression and secretion machinery that can express native and heterologous enzymes, as well as more complex proteins such as human antibodies.^151−155^ A list of enzymes and metabolic pathways expressed in this organism is available in Sun et al.^156^

The availability of a genome sequence combined with the development of molecular and genetic tools have greatly advanced *A. oryzae’s* application in all the above-mentioned research areas. With the availability of transformation systems such as protoplast-mediated, *Agrobacterium*-mediated, and electroporation techniques, genetic manipulation of *A. oryzae* can be successfully achieved, albeit with different transformation efficiencies. These transformation techniques have been aided by the availability of selectable auxotrophic markers such as *pyrG* (orotidine-5′-phosphate decarboxylase), *argB*, *niaD* (nitrate reductase), *sC* (ATRP sulfurylase), *adeA* (phosphoribosylaminoimidazolesuccinocarboxamide synthase), *adeB* (phosphoribosylaminoimidazole carboxylase), and antibiotic markers such as *amdS* (acetamidase), *ptrA* (pyrithiamine-resistant), *thiI*(thiamine transporter), *blmB* (bleomycin N-acetylatransferase) and *AosdhB* (succinate dehydrogenase).^157^ Multiple selection marker techniques such as double, triple and quadruple auxotrophic systems have been developed enabling multigene-editing abilities in this fungus.^46,159,160^ Low homologous recombination (HR) efficiency in *A. oryzae* is a major gene-editing challenge encountered in this fungus, which has been alleviated by the generation of *ku70* and *ku80* gene deletion mutants. Approximately 70% improvement in HR efficiency was observed in the different single and double *Ku* mutants compared to the parental strain.^161^ Disruption of *ligD* gene (*N. crassa mus-53* homologue) has led to ∼90% efficiency in HR frequency, which is even higher than the *ku70*-deficient strain,^162^ thus suggesting other strategies for HR improvements in this fungus. Neither the *ku* nor the *ligD* gene disruptions resulted in any phenotypic defects in this fungus,^161,162^ thereby allowing usage of these deletion mutants in additional strain engineering applications. A loop-out deletion method has also been developed that allows deletion of larger genomic regions in this organism.^110^ This technique, in combination with nonhomologous end-joining deficiency, can result in >470 kb deletion of genomic regions and has been successfully implemented in this fungus.^110^ The Cre-loxP site-specific recombination system has been developed in *A. oryzae,*(163,164) allowing targeted gene or marker editing in this fungus. Lastly, the CRISPR/Cas9 gene-editing system has also been developed for this fungus resulting in improvement in genetic manipulation capabilities.^165^

Despite its immense potential, this fungus has faced hurdles with respect to its development as a chassis microorganism. Due to the absence of a sexual life cycle, deploying conventional genetic manipulations is a challenge with this fungus. This fungus also faces bottlenecks with respect to foreign protein expression. The lack of complete understanding and control of protein trafficking and secretion is a major issue to be resolved in this fungus as well.^157^

#### 2.6.3 *Aspergillus nidulans*

*A. nidulans* is a leading model organism that has been used to study a wide range of topics in biology including enzymology, DNA repair, and primary metabolism, and is the subject of over 5000 research papers.^166^ The organism’s rich collection of biosynthetic gene clusters (BGCs) also makes it an important organism for industrial use in a wide variety of contexts, though it is estimated that approximately half of the BGCs in this organism have still not been conclusively tied to a particular function, as reviewed by Caesar and co-workers.^166^*Aspergillus nidulans* was an early organism used for genetic studies, in part because unlike many *Aspergilli*, it can reproduce sexually and this enables mating crosses.^167^*A. nidulans* has been used to study a wide range of topics in biology including enzymology, DNA repair, and primary metabolism, and its rich collection of BGCs also makes it an important organism for industrial use, though many of the metabolic pathways in this organism remain incompletely elucidated.^166^*A. nidulans* is also promising for the industrial production of enzymes such as aryl oxidase,^168^ cellulase,^169^ and tannase^170^ among others. A thorough review of enzyme production in this organism was recently written by Kumar.^171^ There are also a large number of silent gene clusters in *A. nidulans* that might be useful for making additional important chemical products.^166^

Nonhomologous recombination in this organism has been reduced by a deletion of the *nkuA* gene, which is the homologue of human Ku70.^111,172^ Recently, Vanegas and co-workers developed a CRISPR/Mad7 system that works in *A. nidulans* strains that does not have *nkuA* deleted (i.e., it is NHEJ-proficient).^173^ This suggests that there may be methods of modifying other fungi that do not rely on NHEJ-deficient strains.

Many markers in this organism rely on auxotrophic strains, such as auxotrophs for uridine, uracil, riboflavin, and pyroxidine,^111^ as well as pyrimidine, biotin, lysine, *p*-aminobenzoic acid, and choline.^174^ Resistance to glufosinate conveyed by the *bar* gene^172,175^ and pyrithiamine resistance conveyed by the *ptrA* gene^111^ have also been used as markers.

Transformation of *A. nidulans* can be accomplished using chemically competent cells (i.e., the cell wall of the organism is enzymatically digested to make it more permeable to DNA).^176^ Transformation of *A. nidulans* is frequently done by transforming into protoplasts,^177,178^ and can also be accomplished via *Agrobacterium*-mediated transformations^179^ or particle bombardment.^180^ Similar to other historical strains, *A. nidulans* can have the problem of accumulated mutations which differ from lab to lab.^174^ Studies in *A. nidulans* benefitted greatly from the publication of the full genome of the organism in 2005 by Galagan and co-workers.^148^ The *A. nidulans* genome can be found in a number of different databases such as the Central Aspergillus Data Repository (CADRE)^182^ and FungiDB.^13^ With the availability of the genome, it is possible to find gene clusters using algorithms such as SMURF^183^ and antiSMASH.^184^ This organism has been very extensively researched, and made highly tractable, however the extensive advances made in this organism are beyond the scope of this review. Readers interested in exploring *A. nidulans* further are directed to a number of reviews dedicated to this organism.^166,185−189^

## 3 Genome Engineering Tools

### 3.1 Fusion PCR

Fusion PCR has been a staple in the genetic modification of filamentous fungi, allowing for the rapid production of long homologous fragments useful for efficient recombination. Typically, two primer pairs are designed from sequencing data to amplify two desired regions from genomic DNA. A third primer pair is used to amplify a selectable marker, often sourced from a stable stock like a plasmid, to be inserted between the genomic regions. Finally, a fourth PCR reaction combines the three fragments. The primers for amplifying the selectable marker contain overhanging sequences that facilitate fusion in a third PCR reaction. Specific protocols vary; for instance, some protocols include gel purification of fragments or an additional PCR to amplify enough DNA for subsequent transformation protocols.^190,191^ Other protocols add restriction enzyme recognition sites to the overhanging region of primers, allowing the completion of the 3-way fusion construct through restriction digestion.^190^

Fusion PCR is still widely used in filamentous fungal engineering and construct design, and remains useful for producing inexpensive knockout libraries, such as the work of Zhao and co-workers in *A. fumigatus*,^192^ as well as custom gene targeting inserts.^193,194^ For example, Wei and co-workers used the method to integrate sgRNAs into plasmid vectors for gene-replacement to improve the industrial production of pneumocandin B0 precursor in *Glarea lozoyensis*.^194^ Use of modern high-fidelity polymerases and the availability of cheaper sequencing to validate final constructs has reduced traditional concerns about PCR-induced sequence errors. With intentional design, fusion PCR intermediates and final constructs can be integrated into plasmid toolkits for long-term storage and reuse, and improved shareability between researchers. Fusion PCR bypasses the additional *E. coli* cloning steps, while generating linearized fragments ready for transformation. However, while fusion PCR is effective, it requires at least three PCR reactions to generate the desired construct, and unlike GoldenGate and other genetic toolkits, it does not result in intermediate storable plasmids. Purchase of custom DNA for rapid integration into genetic toolkit part plasmids avoids issues with internal GoldenGate-specific restriction enzyme cut sites present in genome-amplified fragments which limit toolkit compatibility. Custom DNA further allows for codon-optimized fragments to be integrated into genetic toolkits, and to be used more readily in modular cloning approaches. The advancement in gene synthesis technologies have immensely reduced the cost of purchasing custom DNA that may eventually replace the time involved in generating fusion constructs using traditional PCR approaches.

### 3.2 Modular Cloning (MoClo) Tools

**Figure 2:**
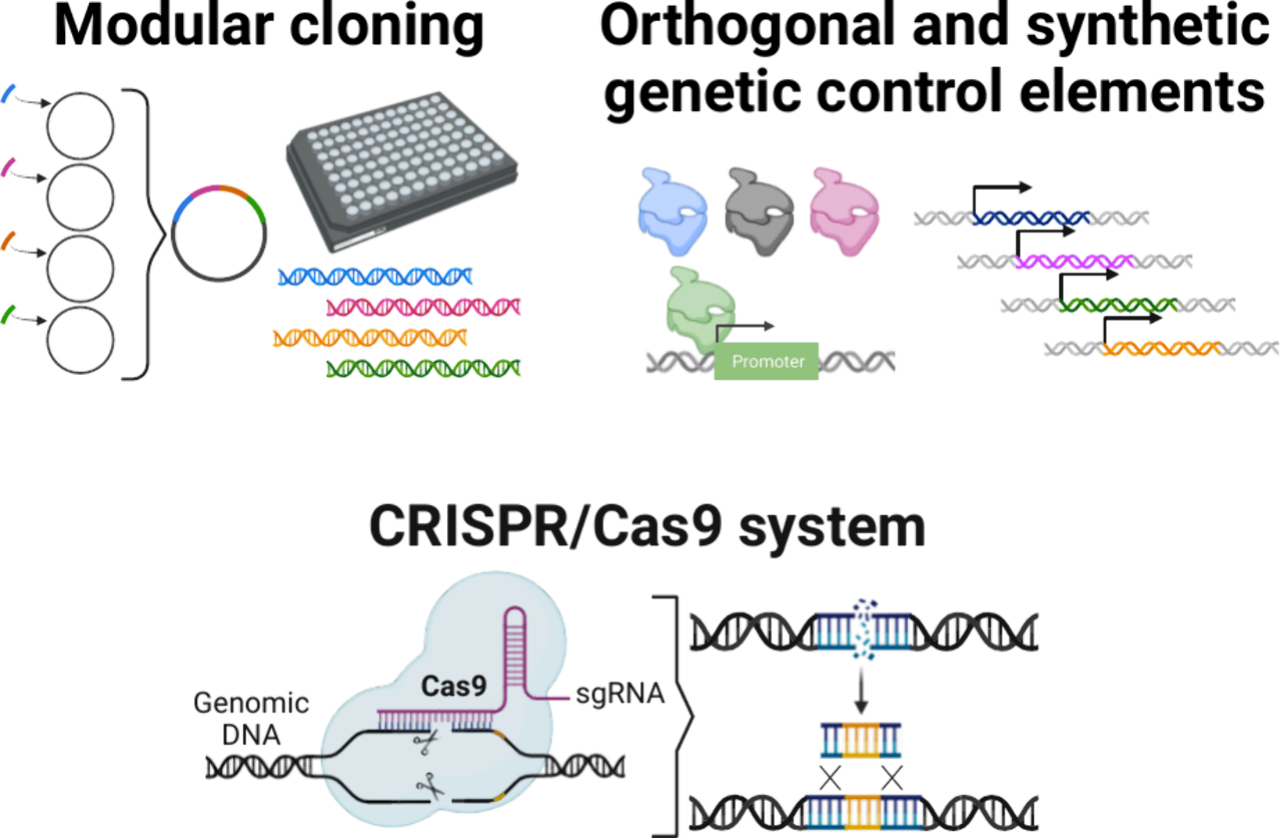
Genome engineering tools. Modular cloning tools have made it easier to build in high throughput large sets of constructs for screening. For example, one pot plasmid construction allows combinatorial assembly on a large scale resulting in a finer resolution of variants that may be screened. Orthogonal and synthetic genetic control elements bring stringent control and dynamic range to expression systems by providing specificity, limiting off-target effects, and expanding control of expression strength. For example, directed evolution experiments result in RNA polymerase variants with improved function and these new polymerases are then added to the toolkit for the target biological system. As another example, screening of promoter libraries results in characterized promoter strengths allowing a tunable range of expression within the target biological system. CRISPR/Cas9 has brought site-specific genome editing to a number of novel biological systems and CRISPR systems are continuing to improve in orthogonality and diversity. Figure created with Biorender.com.

Modular cloning approaches harness the power of combinatorial assembly by facilitating the importation of genetic parts (promoters, ribosome-binding sites, signal peptides, etc.) from diverse natural and synthetic sources into a standardized DNA assembly framework, enabling precise control over the order, directionality, and arrangement of the parts as they are self-assembled in a restriction-ligation step into larger constructs such as transcriptional units (Figure 2). Building large libraries of characterized parts will allow for fine-tuning of complex genetic circuitry. It will also enable experiments directly in support of systems biology efforts to understand the more-global behavior of the cells, as it will be possible to make constructs targeting multiple parts of a pathway simultaneously. Constructing large libraries will also enable high-throughput mutagenesis and screening, as it is an easy way to generate a large number of candidates to screen. The relatively straightforward “plug-and-play” design of modular cloning systems enables even relatively modest laboratories, or technicians with less training, to clone sophisticated constructs, democratizing synthetic biology research in filamentous fungi.

Early attempts to build standardized methods for cloning, NOMAD and BioBricks,^195,196^ were limited to pairwise assembly of parts per reaction and were not very convenient or advantageous to automate. Weber et al. and Sarrion-Padrigones et al. were the first to demonstrate modular and hierarchical DNA cloning systems that enabled assembly of multiple parts (from 2 to >10) in a single step, starting from intact plasmids containing the desired parts.^197,198^ These new systems support the cloning of entire pathways. Weber et al. named their system “MoClo,” a moniker that has since come to refer to other similar methods used in the synthetic biology community. Other standardized methods for DNA assembly have been reviewed elsewhere.^199^

A benefit to hierarchical and modular cloning strategies is the ability to easily assemble genetic constructs for either episomal replication or genomic integration. There are very few persistent replicating vectors for filamentous fungi.^38,200^ Most improvements to episomal expression have been performed in diverse yeast species.^201,202^ Rapid prototyping of genetic parts, including potential plasmid origins of replication, is more realistic in filamentous fungi when using cloning strategies that allow easy genetic part import and integration. Early attempts to build MoClo genetic toolkits for filamentous fungi were limited by constraints on the applicability of the genetic parts and transformation protocols across diverse fungal species.^203^*Agrobacterium*-mediated transformation (AMT) was first used to transform *Saccharomyces cerevisiae* in 1995.^204^ A few years later de Groot et al. reported the use of AMT for transformation of seven filamentous fungal strains including both Ascomycetes and Basidiomycetes.^205^ In 2005, a review by Michielse reported AMT being used on over 50 fungal species.^206^ A 2017 review noted that a search of fungal AMT resulted in more than 900 papers.^207^ AMT can be a useful tool for fungal forward genetic approaches by creating random T-DNA induced mutations as well as reverse genetics and targeting specific genes for replacement or expression changes.^208^ The technique is the subject of two excellent reviews.^209,210^ The GoldenBraid modular cloning toolkit relies on AMT for genetic transformation of plants.^198^ The subsequent expansion of GoldenBraid to include genetic parts for filamentous fungi, known as FungalBraid, significantly advanced the number of parts that could be screened and tested in diverse fungi.^211^ The further expansion of genus-specific modular cloning toolkits, such as TrichoGate for *Trichoderma*(212) will facilitate functional genomics studies, especially in instances where codon bias or expression variation may limit the interchangeability or usefulness of parts in existing MoClo toolkit libraries.

Development of additional fungi as expression systems is quite limited compared to popular expression systems such as *E. coli* and *S. cerevisiae*. Although genome integration is the predominant route for genetic engineering of filamentous fungi, the addition of reliable and long-lasting episomal vectors would increase the speed of rapid prototyping efforts and would thus be a welcome addition to the fungal genetic toolbox. Most recently, Mozsik et al. released a 96-part toolkit for *Penicillium* and *Aspergillus* species, with parts enabling characterization of expression via genomic integration or autonomous vector replication through an AMA1-like sequence.^213^ The AMA1 sequence was originally identified in *Aspergillus nidulans* as part of an extrachromosomal replicating plasmid and is capable of replication in a number of *Aspergillus* species.^214,215^ The availability of new modular toolkit parts for filamentous fungi will make it easier for new research groups to investigate and leverage biological systems of fungi.

### 3.3 Genetic Control and Orthogonal Control Elements

Novel organisms provide an opportunity for accessing novel products or processes that could benefit industry and human health. Application of these novel organisms is limited in part by the lack of robust and predictable gene expression tools for the given species. Classically, natural promoters are identified through genomic and transcriptomic experiments, wherein gene proximity and transcription start sites are carefully mapped. Biochemical and mutational analysis further dissects the architecture of these promoter regions; motifs can be mapped back to specific transcription factors (TFs). The identification and application of suitable promoters for production strain development benefits from a large body of work devoted to understanding the design parameters of promoters in different organisms. The importance of strong and inducible promoters for industrial production strains is reviewed elsewhere.^216,217^ However, species-specific genetic controls take time to characterize and develop, which creates a bottleneck in implementing novel organisms for production purposes. Even with the use of species-specific and broad host control elements, interference with the endogenous system may stymie further engineering efforts. Therefore, it would be attractive to develop synthetic and orthogonal gene elements that could be used as universal promoters across multiple fungal species, as this would not require extensive retuning of organisms, and the orthogonality would eliminate the interference that may be caused by systems similar to that of the host species.

A synthetic TF, with a tailored modular promoter system, was developed in *S. cerevisiae*, and it was demonstrated to promote a range of predictable expression across a number of growth conditions.^218^ The synthetic expression system was refined, and its universality was demonstrated in diverse fungi including *Pichia (Komagataella) pastoris, Pichia kudriavzevii*, *Yarrowia lipolytica, Candida apicola*, *Zygosaccharomyces lentus, A. niger,* and *T. reesei.*(219) This universal and orthogonal expression system circumvents the bottlenecks of species-specific expression control and interference with native regulation.

Synthetic promoters previously were designed computationally for several yeast species.^220−223^ The conservation of promoter architecture across eukaryotes suggests that these tools can be applied to more diverse fungi. The primary difficulty in screening synthetic promoter libraries in fungi, particularly filamentous fungi, stems from the low-throughput and low efficiency of transformations, a problem that we have discussed elsewhere.

One orthogonal promoter that has been the subject of recent efforts in filamentous fungi is the T7 promoter, derived from T7 bacteriophage. This is a simple and strong orthogonal transcription system that has been widely used in prokaryotic synthetic biology (reviewed in^224^). The T7 system has been used for heterologous expression *in vitro* and *in vivo*, including in eukaryotes, however the T7 system faces difficulties in eukaryotes due to post-transcriptional processing of mRNA (mRNA), required for export from the nucleus and protein synthesis.^225^ T7 RNA polymerase has been used with limited success *in vivo* in *A. fumigatus* as a prototype RNA interference method.^226^ Recent work in *S. cerevisiae* demonstrates that T7 promoter-driven protein expression can be improved using viroporins, increasing the amount of T7 transcript entering the cytoplasm, thereby increasing protein production from T7 (uncapped) mRNA.^227^ The versatility of the T7 system, and its wide use in engineered prokaryotes, suggests that efforts should continue to bring this system into wider use in filamentous fungi.

The development of further orthogonal and synthetic promoters would benefit from a combined synthetic and systems biology approach, coupled with thoughtful use of available genomic databases. A systems biology study would reveal if the promoter is truly orthogonal, or whether introduction of the promoter is creating some disturbance to the organism that may not be readily apparent. A systems biology approach may also reveal what is causing an orthogonal system to not function well in the eukaryote of interest (e.g., post-transcriptional processing), and thereby identify the necessary modifications to the fungal strain. The identification of natural promoters in genomic databases would provide candidates for development into orthogonal and synthetic gene control elements with universal applicability.

### 3.4 CRISPR Gene Editing

The clustered regularly interspaced short palindromic repeats (CRISPR) RNA-guided nucleases Cas9 and Cas12a (also known as Cpf1) specifically bind and cleave a user-selectable target DNA sequence, resulting in double strand breaks (DSB) which are then repaired by the cell through HR or NHEJ (Figure 1).

Two different strategies to deliver Cas9 endonuclease and the single-guide RNA (sgRNA) into fungal cells have been demonstrated. The first approach includes expressing the Cas9 protein and the guide RNA (gRNA) transcripts *in vivo* by transformation of the respective gene sequences into the cells.^228−230^ The second approach involves cotransformation of intact Cas9 protein and *in vitro* transcribed sgRNA transcript as assembled ribonucleoprotein complexes into the cells, thereby alleviating the challenges associated with constitutive expression of Cas9 in cells.^219,231,232^

Several excellent reviews cover the breakthroughs and challenges of CRISPR technology applied to filamentous fungi.^233−239^ The application space for advancements made using CRISPR-mediated genetic modifications in filamentous fungi includes the study of plant and human pathogens (reviewed in^5^), silent gene clusters for drug discovery,^240^ and improvement of product yields from engineered enzymes or pathways. Availability of this gene-targeting tool is critical to biotechnology, in particular, the engineering of protein producers such as *T. reesei* and *Aspergillus spp*. CRISPR technology is described in other ascomycetes such as *Pyricularia oryzae,*(241)*Neurospora crassa,*(242)*Candida albicans,*(243)*Cordyceps militaris,*(244)*Penicillium chrysogenum,*(232)*Alternaria alternata,*(245)*Nodulisporium spp.,*(246) and *Beauvaria bassiana,*(247) as well as basidiomycetes such as *Ganoderma lucidum,*(248,249)*Ustilago maydis,*(250)*Coprinopsis cinerea,*(244,251) and *Schizophyllum commune.*(252) The ability to harness the power of CRISPR for facile, rapid, and precise genome editing requires control over the timing and expression level of CRISPR system components. As reviewed in Song et al., difficulties with achieving sufficient control over this expression has limited the universality of CRISPR-based genome editing across fungal species. The diversity of components used to apply CRISPR successfully across 34 unique filamentous fungi species attests to the limitations in the reusability of parts and the difficulty in tuning expression across diverse species.^238^

The human-optimized version of Cas9 does not function in *T. reesei* strains, whether expression is driven by a strong constitutive promoter^253^ or an inducible promoter.^254^ Therefore, Liu et al. relied on a codon-optimized Cas9 fused to a reporter in order to validate expression and localization in *T. reesei.*(229) That work also demonstrated multigene targeting using the CRISPR/Cas9 system, although the efficiencies of targeted genome edits were quite low. Notably, genetic manipulations mediated by CRISPR/Cas9 are up to 100% efficient for single gene modifications, compared to 63% efficiency using HR in NHEJ- deficient strains of *T. reesei.*(63)

The first reported example of CRISPR in *Aspergillus* was a 2015 research article published by Nodvig et al., which described successful gene-specific targeting in six species of *Aspergillus*. This work, which combined AMA1-based autonomously replicating vectors, recyclable markers, strong expression components, and creation of the Optimus script (which identifies PAM-protospacer motifs for homologous genes across species) remains state-of-the-art.^255^ In addition, the USER cloning method used for plasmid construction in this work is amenable to rapid one-step assembly of multiple parts for combinatorial screening. Nodvig et al. reported up to 90% gene-targeting efficiency in *A. nidulans*, but the efficiency varied widely across species when a single PAM-protospacer sequence identified with the Optimus script was used.^255^ This result exemplifies some of the difficulties in building genetic toolkits with parts that function well across even closely related filamentous fungal species.

Song et al. demonstrated a strategy for adapting CRISPR into a given *Aspergillus* species using tRNAs (tRNAs) to express and process the sgRNAs. This work used native *A. niger* tRNAs to recruit RNA Polymerase III and to express sgRNAs from an AMA1 plasmid, yielding a mutation efficiency as high as 97%.^256^ When applied to recombineering, this sgRNA expression scheme resulted in 42% integration efficiency in a strain with an intact NHEJ pathway, and >90% efficiency with NHEJ disrupted.^256^ The robust constitutive expression provided by tRNAs makes them a valuable tool for driving expression of sgRNAs, and their self-splicing by native tRNA processing enables them to act as insulating spacers, enabling multiplexing of sgRNA expression,^257^ including in *Aspergilli.*(258)

Classical, sequential genome modifications in filamentous fungi are laborious and time-consuming. CRISPR can provide a fast and efficient route to multiple, simultaneous genome modifications, which is particularly beneficial in species for which a limited number of selectable markers is available. Other factors affecting the application of CRISPR technology in fungi include locus biases, transformation efficiencies, and both nuclease and gRNA expression levels.

Synthetic biology is improving and expanding the use of CRISPR technology in filamentous fungi whose industrial importance continues to increase based on current biotechnology trends. Sarkari et al. used AMA1-based plasmids for transient expression of Cas9 to improve titers of aconitic acid from engineered strains of *A. niger.*(259) Use of a split selection marker reduced the incidence of false-positive transformants. Further, modular cloning frameworks facilitate the construction of fungal vectors.^259^ The dual challenges of collecting and assembling numerous parts for tuning gene expression as well as the often laborious and lengthy process of screening transformants for the desired genotype and phenotype hinder rapid turnaround in fungal strain development for biotechnology. The lack of genetic parts and vectors that can be applied across filamentous fungal species represents a major challenge for synthetic biology when access to diverse organisms is desired. However, the steady increase of engineering strategies applied in filamentous fungi suggests that the scientific community will overcome these challenges and bring fungal chassis organisms to the forefront of biotechnology.

## 4 Advantages of Filamentous Fungi as Chassis Organisms

### 4.1 Microbial Cell Factories

Microbial cell factories leverage biological systems for the manufacture of valuable commodity products. The product portfolio of filamentous fungi, comprising mainly proteins, small molecule metabolites, and biomass is estimated at several billion dollars per year. Products made with fungi are diverse and impact the food, pharmaceutical, construction, textiles, agriculture, and biofuels industries.^260^ There are many aspects of strain development, production, and scale-up that need to be considered when selecting a microbe for commercial or industrial level production of commodity products, such as 1) the metabolic burden of biosynthesis, 2) whether and how enzyme expression and function can be optimized for biosynthesis, and 3) if the chassis organism is amenable to scaled-up production. There are many recent reviews of microbial cell factories.^261−264^ A few species of filamentous fungi are well-known as industrial workhorses (belonging to genera *Aspergillus, Trichoderma, Penicillium, Neurospora,* and *Fusariaum*) and are exploited for their natural metabolic products. However, heterologous expression in these strains remains challenging, for reasons outlined in this review. Although microbes like *E. coli, Bacillus subtilis,* and *S. cerevisiae* are attractive because they have established synthetic biology tools, have been analyzed with systems biology approaches, and have proven scalable for commercial production of various biomolecules, the natural capability of filamentous fungi to utilize diverse feedstocks, their unique metabolic processes, and secretion of enzymes makes them very attractive as chassis for production.

### 4.2 Expression of Heterologous Biosynthetic Gene Clusters (BGCs) in Chassis Organisms

Filamentous fungi are of interest for their ability to express a wide variety of proteins, and synthesize a wide range of small molecules. They have certain advantages over simpler organisms such as yeast because of their ability to express complex protein clusters. Heterologous expression is a strategy that can be used to prevent metabolic burden when one or multiple components of a native system are overexpressed. A desired product can be made via heterologous expression after the identification, characterization, and expression of the appropriate BGCs. For example, Han et al. used sequence similarity networks analysis^265^ to mine the genome of the basidiomycete *Hericium erinaceus* for enzymes potentially involved in the synthesis of orsellinic acid, belonging to a class of compounds with vast pharmaceutical potential.^266^*A. oryzae*, an ascomycete, was used for heterologous expression of the seven-intron containing *herA* gene leading to the production of orsellinic acid.^266^ This example of heterologous expression is noteworthy for the use of an ascomycete to correctly splice and translate a complex basidiomycete gene for the goal of natural product discovery, circumventing the need to culture the origin basidiomycete for scale-up production, a feat impossible for lower microbes. *A. oryzae* was also used in the production of a functional human antibody, suggesting that this organism has potential as a chassis for the production of complex, biomedically relevant proteins.^155^

Using high throughput approaches, genome mining identifies active BGCs based on available systems biology data and finds silent or cryptic BGCs based on predictive models and homology. As the number of filamentous fungal genomes grows, novel BGCs will be characterized. Active BGCs can be directly interrogated in the native species or heterologously expressed. Cryptic BGCs may be difficult to interrogate in the native species as this requires precise genome modifications to turn on expression.^240^ The choice of chassis for heterologous expression to explore natural products produced by novel BGCs will depend on the origin host and whether synthetic biology tools are available for a closely related species able to fulfill post-translational modifications of expressed proteins.^267^ Entire secondary metabolite gene clusters can be expressed in filamentous fungi such as *A. nidulans* at high yield, further showing the advantages of these types of organisms compared to simple yeasts.^111^ Using modular cloning methods such as MoClo, it should be possible to clone isolated systems of genes into appropriate chassis organisms, which enables experimental study of how those genes work together in isolation from other connections that might occur in their native hosts.

Currently, filamentous fungi are more often the source of BGCs, rather than the host for heterologous expression of them. Liu et al. suggested three strategies to streamline natural product discovery from fungal BGCs: 1) optimize *S. cerevisiae* for fungal BGC expression 2) streamline BGC identification and 3) optimize high throughput cloning of BGCs.^268^ For example, the refactoring of a plant biosynthetic pathway into *S. cerevisiae* to produce monoterpene indole alkaloids required 56 genetic edits, including heterologous expression of 34 plant genes and modification of 10 yeast genes to improve native metabolic flux toward the biosynthetic pathway of interest.^269^ However, not all biosynthetic pathways or BGCs in filamentous fungi can be heterologously expressed in yeast, meaning filamentous fungi chassis are required to fully explore the ever-growing number of fungal BGCs (reviewed in^270^). Even when heterologous expression of BGCs in *S. cerevisiae* is successful, the yields are often very low, and careful attention must be paid to the yields reported in the literature.

### 4.3 Morphology and Fermentation Conditions

In general, the growth rate of filamentous fungi is slower than that of *E. coli* and yeast, which is often a contributing factor when deciding on a chassis for scale-up. Growth and morphology dramatically impact yields of metabolite, protein, or chemical production from cultures (reviewed in^271^), and wild strains often have a high secondary metabolite background, resulting in many peaks in analytical techniques such as LC-MS, which makes it difficult to identify and isolate metabolites of interest. Heterologous expression in low background strains can help reduce or eliminate this problem. The growth phenotype is influenced in industrial applications by factors such as ion concentrations,^272^ carbon source,^273,274^ or pH^275,276^ to name a few. In some cases, industrial workhorse strains have been engineered to be more resistant to stresses imposed by at-scale production culture conditions. For example, Cai et al. knocked out polarized growth genes in *Aspergillus glaucus*, which conferred shear-resistance and thereby increased the production rate of the antitumor polyketide aspergiolide.^277^ Large-scale fermentation can also be improved by reducing the propensity of certain filamentous fungi to form hyphal pellets. Preventing the formation of these hyphal pellets improves the dispersion of the fungus in a fermenter, resulting in higher yields. In order to accomplish this, Miazawa and co-workers targeted the genes responsible for synthesis of α-1,3-glucans in the cell walls of *A. oryzae* and *A. nidulans*, with the deletion of α-1,3-glucan genes being sufficient to allow complete dispersion of *A. nidulans* and the formation of smaller pellets in *A. oryzae*, with corresponding increases in yield.^278,279^ In a follow-up study, the authors found that an additional deletion of galactosaminogalactan (GAG) genes prevented pellet formation in *A. oryzae,* and resulted in improved yield.^280^ Genes for α-1,3-glucan or GAG biosynthesis are found in many fungal species,^281^ meaning that targeting these genes may produce improvements in the dispersion of these fungi in liquid fermenters and therefore increase yields. The complexity of the filamentous fungal lifecycle can complicate optimization of product yields, but careful process engineering combined with metabolic engineering can overcome the challenges in optimizing production.

Classically, metabolic engineering involves random mutagenesis, but synthetic biology offers a more sophisticated approach to manipulating carbon flux for chemical production (reviewed in^37,202^). Those approaches have been applied successfully to the breakdown of plant biomass to useful products (reviewed in^282,283^).

The application of systems biology approaches in conjunction with synthetic biology can also result in higher titer and yields. For example, Nakamura and Whited used systems biology to identify genes in the metabolic pathway of 1,3-propanediol. After designing a pathway, they isolated the needed genes from various naturally producing strains and engineered an industrial strain of *E. coli* to produce the target compund from glucose.^284^ Similarly, Yim et al. used predictive computational modeling to identify a pathway for 1,4-butanediol and balance the energy and redox needs of the engineered *E. coli*.^284,285^ Developing and deploying similar tools and approaches to filamentous fungal systems could have a major impact on the production of industrial chemical, pharmaceutical, antibiotic, and other bioproducts.

In US patent US11028401B2, Bruno and co-workers at Zymergen reported the use of laboratory automation equipment and workflows to accelerate and increase the throughput of genetic manipulation for strain engineering of *A. niger* by first identifying the genotypes of existing strains with preferred morphological properties.^286^ The *A. niger* type strain ATCC 1015 produces about 7 g of citric acid from 140 g of glucose. A descendant of ATCC 1015 strain named ATCC 11414 was selected in 1946 and exhibits substantially increased citric acid production (70 g) under the same conditions.^286,287^ The Zymergen patent describes genome sequencing of both strains, leading to the identification of 43 single nucleotide polymorphisms between the two genomes.^286^ A primary goal of this effort was to discover genetic determinants of hyphal morphology to engineer strains of *A. niger* with “non-mycelium, pellet morphology” that result in submerged cultures with lower-viscosity and higher rates of mass transfer for oxygen and nutrients, which are desirable for large-scale cultivation in fermentation tanks. Currently, it is not possible to precisely predict the growth morphology for optimal product yield, and so current work requires investment in process design.^271^

## 5 Perspective on Scaling up for Biomanufacturing

As the ability to screen in high throughput becomes more accessible for filamentous fungi, one thing that needs to be considered is the replication of bioreactor conditions, as laboratory-scale and bioreactor-scale production can often be very different. New “microchemostat” screening technologies such as Beacon system allow simultaneous culture of ∼10^5^ segregated colonies of differing genotypes under conditions mimicking a bioreactor.^288^ However, microchemostat screening has yet to be applied to filamentous fungi.

An important factor in bioreactor-scale production is phenotypic heterogeneity, which is implicit to the “survival of the fittest” paradigm. In nature, phenotypic heterogeneity is an advantage, whereas in fixed environments, such as a bioreactor or fed-batch production, it can be detrimental to the end-product.^289−291^ Phenotypic instability manifests from genetic mutations in the nucleus or mitochondria, epigenetic mutations, and variability of unknown cause whose incidence increases dramatically with the number of cell divisions. Lopez et al. evaluated the impact of engineered *S. cerevisiae* strain design and construction on production performance and scalability. They found that the engineered strain producing higher yields of β-carotene under shake-flask conditions was outperformed by a second engineered strain under fed-batch conditions, indicating that production characterization can be misleading if not performed under actual production conditions.^292^ They further characterized genetic instability due to integration architecture, which is a disadvantage necessitating prebioreactor screening of colonies; however the issue was resolved through another design-build-test round.^292^ This example highlights the need for characterizing strains for scale-up for both genetic stability and performance under production conditions, which will be important as filamentous fungal chassis are brought up as microbial cell factories.

The ideal chassis will have reduced susceptibility to toxic intermediates, optimal growth under required conditions, and need limited refactoring or have readily available tools to do so. The final engineered strain should then demonstrate enough genetic and phenotypic stability to limit any effects during fed-batch culture and have good yields of product at fed-batch scale. The iteration of strain engineering and process engineering is necessary for optimal performance and yields, but is beyond the scope of this review.

## 6 Conclusion

The multitude of enzymes, metabolic pathways, and intermediates encoded in the DNA of fungi are only beginning to be understood and are important resources that could be harnessed for a broad range of applications. While some genetic tools are available to make filamentous fungi viable for production applications, and there are certain workhorse organisms such as *A. oryzae*, *N. crassa*, and *A. nidulans*, development of facile genetic systems in additional organisms is still needed, which could be challenging given the diversity of fungi. Once developed, the genetic tools can be used to elucidate the fundamentals of an organism (connecting genotype to phenotype), improve production of target molecules in organisms, and create synthetic organisms that use DNA programming to combine functions from multiple species. Conversion of filamentous fungi to synthetic biology chassis organisms would allow many of the inherent benefits of the organisms (robustness, metabolic diversity, etc.) to be leveraged when adding synthetic pathways.

The future of fungal genetic systems and synthetic biology in filamentous fungi for biotechnology is promising. Genetic tools are constantly in development for prokaryotic and eukaryotic systems, and systems biology studies are building the level of understanding required to begin designing synthetic systems. While the tools in their native format may not work in filamentous fungi, they have the potential to be adapted to fungal hosts. The combination of increased understanding of under-studied organisms, improved predictive models, better tools for genetic manipulation, and cheaper and more modular assembly of DNA circuits is precisely what is needed to advance the use of filamentous fungi in synthetic biology.

## Abbreviations

AMT: *Agrobacterium*-mediated transformation
CRISPR: clustered regularly interspaced short palindromic repeats
DSB: double strand break
FACS: fluorescence activated cell sorting
FADS: fluorescence activated droplet sorting
gRNA: guide ribonucleic acid
HR: homologous recombination
MoClo: modular cloning
mRNA: messenger RNA
NHEJ: nonhomologous end joining
sgRNA: single-guide ribonucleic acid
TF: transcription factor
tRNA: transfer RNA
MCF: microbial cell factory
BGC: biosynthetic gene cluster
